# Snail knockdown reverses stemness and inhibits tumour growth in ovarian cancer

**DOI:** 10.1038/s41598-018-27021-z

**Published:** 2018-06-07

**Authors:** N. Hojo, A. L. Huisken, H. Wang, E. Chirshev, N. S. Kim, S. M. Nguyen, H. Campos, C. A. Glackin, Y. J. Ioffe, J. J. Unternaehrer

**Affiliations:** 1 Division of Biochemistry, Department of Basic Sciences, Loma Linda University, Loma Linda, CA USA; 2 Department of Molecular Biology, Chonbuk National University, Dukjindong 664-14, Jeonju, Jeollabuk-do, 561-756 Republic of Korea; 3 University of California, Riverside - School of Medicine, Riverside, CA USA; 4 Center for Health Disparities and Molecular Medicine, Loma Linda University, Loma Linda, CA USA; 5 Beckman Research Institute, City of Hope, Duarte, CA USA; 6 Division of Gynecologic Oncology, Department of Obstetrics and Gynecology, Loma Linda University Medical Center, Loma Linda, CA USA

## Abstract

To develop effective therapies for advanced high grade serous ovarian cancer (HGSOC), understanding mechanisms of recurrence and metastasis is necessary. In this study, we define the epithelial/mesenchymal status of cell lines that accurately model HGSOC, and evaluate the therapeutic potential of targeting Snai1 (Snail), a master regulator of the epithelial/mesenchymal transition (EMT) *in vitro* and *in vivo*. The ratio of Snail to E-cadherin (S/E index) at RNA and protein levels was correlated with mesenchymal morphology in four cell lines. The cell lines with high S/E index (OVCAR8 and COV318) showed more CSC-like, motile, and chemoresistant phenotypes than those with low S/E index (OVSAHO and Kuramochi). We tested the role of Snail in regulation of malignant phenotypes including stemness, cell motility, and chemotherapy resistance: shRNA-mediated knockdown of Snail reversed these malignant phenotypes. Interestingly, the expression of let-7 tumour suppressor miRNA was upregulated in Snail knockdown cells. Furthermore, knockdown of Snail decreased tumour burden in an orthotopic xenograft mouse model. We conclude that Snail is important in controlling HGSOC malignant phenotypes and suggest that the Snail/Let-7 axis may be an attractive target for HGSOC treatment.

## Introduction

Metastatic chemotherapy-resistant tumour recurrence is the primary cause of the 70% five-year mortality observed in patients with advanced high grade serous ovarian cancer (HGSOC). Despite successful initial surgery and chemotherapy, most patients will experience recurrence of their disease, and of those who do, most will not respond to treatment with conventional chemotherapy modalities^1–4^. We aim to understand mechanisms of recurrence and metastasis, toward the goal of developing targeted therapies. We have focused our efforts on HGSOC which causes 90% of ovarian cancer deaths.

Epithelial-mesenchymal transition (EMT), the process by which cells gain the ability to exit the epithelial layer and invade through the basement membrane, occurs as part of normal embryonic development^5^. In cancer, transitions between epithelial and mesenchymal states are involved in processes leading to the aggressive phenotype that allows cells to leave the primary tumour, invade secondary sites, and form metastases^6^. Of the transcription factors controlling EMT, we chose to focus on Snai1 (Snail) because its expression is sufficient for EMT^7^, it transcriptionally activates pluripotency-related genes^8^ and its expression has been linked to stem cell characteristics in several cancers including breast^9,10^, liver^11^, ovarian^12^, colorectal^13^, and squamous cell carcinoma of the head and neck^14^. Snail expression increases as HGSOC advances^15,16^, and correlates with poor prognosis^17^. Expression of the transcription factor Snail (SNAI1) causes EMT by repressing the transcription of E-cadherin (CDH1), an adherens junction protein important for epithelial phenotype^7^, and other epithelial factors. With the loss of E-cadherin there is a switch to N-cadherin (CDH2) production in the mesenchymal phenotype^18^.

In addition to its actions leading to EMT, Snail has other effects in normal epithelial cells, including growth arrest and resistance to apoptosis^19^. Partial EMTs, leading to cells expressing both mesenchymal and epithelial markers (E/M, also known as the hybrid phenotype), may also be relevant for the acquisition of stem cell characteristics^20^. The hybrid state has been associated with aggressiveness and poor outcome in ovarian and other cancers^21–23^. These E/M cells have been identified in primary tumour samples, and are the cells responsible for xenograft formation^21^.

Expression of Snail has been linked to acquisition of stem cell characteristics^8,9,24,25^. We have suggested that one mechanism by which Snail leads to stemness is by inhibiting let-7, a miRNA that maintains the differentiated state. We demonstrated that Snail binds promoters of miRNA Let-7 family members during the process of reprogramming somatic cells to pluripotency^25^. Let-7 promotes differentiation and inhibits self-renewal via its targets including HMGA2, LIN28, IMP-1, CDC34, and many others;^26^ it is expressed in somatic cells and absent in pluripotent cells. Let-7 acts as a tumour suppressor due to its repression of targets such as c-Myc and Ras^27^. Let-7 is lost in many cancers, including ovarian^28^.

Careful selection of cell lines facilitates their use as phenotypically accurate and thus clinically useful *in vitro* models of HGSOC. Very few publications have reported on the HGSOC cell lines with the highest degree of fidelity to patient samples^29^. In this work, we describe HGSOC cell lines that accurately reflect the gene expression signature of HGSOC patient samples. Epithelial and mesenchymal characteristics of these cells are described, focusing on the master EMT regulator Snail. Based on the EMT perspective, we correlate the presence of mesenchymal state with stem cell markers and function.

## Materials and Methods

### Cell cultures

OVSAHO, Kuramochi, and COV318 human ovarian cancer cell lines were the kind gift of Gottfried Konecny (University of California Los Angeles), OVCAR8 human ovarian cancer cell line from Carlotta Glackin (City of Hope), D2F human fibroblast cell line and NCCIT embryonal carcinoma cell line from George Daley (Harvard Medical School). OVSAHO, OVCAR8 and D2F cells were cultured in Dulbecco’s Modification of Eagle’s Medium (DMEM) with 10% fetal bovine serum (FBS), 2 mM of L-Glutamine, 100 U/mL of penicillin, and 10 μg/mL of streptomycin. COV318 cells were cultured in DMEM with 10% FBS, 2 mM of L-Glutamine, 100 U/mL of penicillin, 10 μg/mL of streptomycin, and 0.25 μg/mL of Gibco Amphotericin B. Kuramochi cells were cultured in Roswell Park Memorial Institute Medium (RPMI) with 10% FBS, 2 mM of L-Glutamine, 0.25 U/mL of human insulin, 1x MEM non-essential amino acids (NEAA), 100 U/mL of penicillin, 10 μg/mL of streptomycin, and 0.25 μg/mL of Gibco Amphotericin B. NCCIT cells were cultured in RPMI with 10% FBS,2 mM of L-Glutamine, 1 mM of Sodium pyruvate, 1X NEAA, 100 U/mL of penicillin, and 10 μg/mL of streptomycin.

### Real-time quantitative reverse-transcription PCR (qRT-PCR)

Total RNA from cell culture samples was isolated using TRIzol reagent (Life Technologies, Carlsbad, CA, USA) according to the manufacturer’s instructions. For mRNA expression analysis, cDNA was synthesized with 1 μg of total RNA using Maxima First Strand cDNA Synthesis Kit (K1672; Thermo fisher scientific, Grand Island, NY, USA). Real-time qRT-PCR for mRNA was performed using PowerUP SYBR Green master mix (Thermo fisher scientific, Grand Island, NY, USA) and specific primers on a Stratagene Mx3005P instrument (Agilent Technology, Santa Clara, CA, USA). The sequence of primers for mRNA quantitation is shown in Table S1. For miRNA expression analysis, cDNA was synthesized with 100 ng of total RNA using specific stem-loop RT primers and TaqMan microRNA Reverse Transcription Kit (Applied Biosystems, Foster City, CA, USA). Real-time qRT-PCR for miRNA was performed using TaqMan Universal PCR Master Mix II (Applied Biosystems, Foster City, CA, USA) with specific primers and probes on a Stratagene Mx3005P instrument (Agilent Technology, Santa Clara, CA, USA). The primers and probes for miRNA quantitation were supplied with the TaqMan microRNA Assay (Applied Biosystems, Foster City, CA, USA). The results were analysed using the ΔΔ cycles to threshold (ΔΔCt) method.

### Western blot

Cells were lysed, and proteins were separated by SDS-PAGE and transferred to PVDF membrane. After blocking of non-specific binding, immunoblots were incubated with primary antibodies for Snail (L70G2; Cell Signaling Technology, Danvers, MA, USA)^30^, E-cadherin (610182; BD Biosciences, San Jose, CA, USA)^31^, α/β-tubulin (2148 S; Cell Signaling Technology, Danvers, MA, USA)^32^, and GAPDH (14C10; Cell Signaling Technology, Danvers, MA, USA)^33^ followed by incubation with an anti-mouse IgG conjugated with DyLight 800 (SA5-10176; Invitrogen, Carlsbad, CA, USA)^34^ or anti-rabbit IgG antibody conjugated with DyLight 680 (35569; Invitrogen, Carlsbad, CA, USA)^35^. Immunoblots were scanned and visualized using Odyssey Infrared Imaging System (LI-COR Biosciences, Lincoln, NE, USA). Densitometry was performed on scanned immunoblots by ImageJ.software (National Institutes of Health, Bethesda, MD, USA).

### Snail/E-cadherin index

Snail/E-cad index (S/E index) was determined based on protein expression levels quantified by Western Blot or mRNA expression levels measured by qRT-PCR. For calculation, the following formula was used:

$$S/E\,{\rm{Index}}={\rm{Snail}}\,{\rm{expression}}\,\mathrm{level}/E \mbox{-} {\rm{cadherin}}\,{\rm{expression}}\,{\rm{level}}$$

### Flow cytometry

Cells in FACS Stain (phosphate-buffered saline (PBS) with 1% FBS, 0.1% Sodium Azide, and 2 mM EDTA) were labeled with antibodies at 4 °C for 15 minutes, washed and then fixed in FACS Fix (FACS Stain with 1% PFA). UltraComp eBeads (01-2222; Thermo fisher scientific, Grand Island, NY, USA) stained with each antibody were used for compensation. Flow cytometry was performed on MACSQuant Analyzer 10 (Miltenyi Biotec, Auburn, CA, USA) and analysis of data was performed using FlowJo Version 10 (FlowJo LLC, Ashland, OR, USA). Fluorescent dye-conjugated antibodies for CD44 (561292; BD Horizon, BD Biosciences, San Jose, CA, USA), CD117 (c-Kit) (130-099-325; Miltenyi Biotec, Auburn, CA, USA), CD133 (130-090-854, Miltenyi Biotec, Auburn, CA, USA), E-cadherin (CD324) (130-099-141, Miltenyi Biotec, Auburn, CA, USA) and N-cadherin (CD325) (563435; BD Pharmingen, BD Biosciences, San Jose, CA, USA) were used.

### Spheroid formation assay

Cells were plated at a density of 5 × 10^4^ cells per well in 6-well non-tissue culture coated plates and maintained in serum-free medium for 7 days. The number of spheroids was counted and statistically analysed. Phase contrast images of spheroids were taken and analysed using ImageJ software (National Institutes of Health, Bethesda, MD, USA) to assess the size of spheroids.

### Scratch assay (Wound-healing cell migration assay)

Cells were grown to 90% confluency in 6-well tissue culture plates then treated with mitomycin C and scratched with a 200 μL micropipette tip. Pictures of fixed positions in the wounds were taken every 4 hours for a 24-hour period with a bright field microscope with phase contrast. The wound area in each picture was measured by ImageJ software (National Institutes of Health, Bethesda, MD, USA).

### Cell growth inhibition assay

A 3-(4,5-dimethylthiazol-2-yl)-2,5-diphenyltetrazolium bromide (MTT, Sigma-Aldrich, St. Louis, MO, USA) assay was used to determine cell viability. Cells were seeded at a density of 1 × 10^3^ cells/well in 96-well plate and incubated overnight. The cells were then treated with increasing concentrations of Cisplatin (0, 0.39, 0.78, 1.56, 3.13, 6.25, 12.5, 25, 50 and 100 μM) for 3 days. After drug treatment, 10 μl of MTT solution was added to each well, and the plates were incubated for 4 h at 37 °C. The formed formazan crystal was dissolved in dimethyl sulfoxide (DMSO), and the absorbance was measured at 570 nm using a SpectraMax i3x microplate reader (Molecular Devices, Sunnyvale, CA, USA). The half-maximal inhibitory concentration (IC50) of cisplatin were analysed using the GraphPad Prism Version 7.0 (GraphPad Software, La Jolla, CA, USA).

### Lentiviral short-hairpin RNA (shRNA) construction and cell transduction

Bacterial glycerol stock containing lentivirus plasmid vector pLKO.1-puro with shRNA specific for SNAI1 (shSnail; SHCLNG-NM_005985) was purchased from Sigma-Aldrich (St. Louis, MO, USA). Scramble shRNA (shScr; Plasmid #1864) was purchased from Addgene (Addgene, Cambridge, MA, USA). Lentivirus particles were produced in HEK293T cells after co-transfection of lentivirus plasmid vector shSnail or shScr with packaging plasmids using X-tremeGene9 (Sigma-Aldrich, St. Louis, MO, USA). After 48 h and 72 h medium containing lentivirus was collected and filtered through 0.22 μM filter. Filtered virus containing medium was used for cell transduction or stored at −80 °C. Cells were transduced with lentivirus in the presence of 6 μg/ml protamine sulfate and selected with puromycin for 4 days.

### Mice

All animal procedures were conducted according to animal care guidelines approved by the Institutional Animal Care and Use Committee at Loma Linda University. Nude mice (nu/nu) were obtained from Jackson Laboratory (Sacramento, CA, USA), were housed in specific pathogen-free conditions, and were used for xenografts at 6–10 weeks of age.

### Cell preparation for xenograft

To allow *in vivo* visualization, OVCAR8 cells were transduced with a CMV-p:EGFP-ffluc pHIV7 lentiviral vector (eGFP-ffluc), which encodes a fusion protein of GFP and firefly luciferase^36^. The eGFP-ffluc-transduced OVCAR8 cells (OVCAR8-ffluc) were selectively isolated based on GFP expression via FACSAria cell sorter (BD Biosciences, San Jose, CA, USA) and then transduced with lentivirus containing shRNA targeting Snail (shSnail) or scramble control (shScr) for xenograft experiment. Lentivirus production and cell transduction were performed by the same procedure described in “Lentiviral short-hairpin RNA (shRNA) construction and cell transduction” section.

### Orthotopic xenograft model and live imaging

shScr- or shSnail-expressing OVCAR8-ffluc cells were injected into the ovarian bursa of nude mice at 1:1 with Matrigel (354248; Corning, Corning, NY, USA) at 2.5 × 10^5^ cells per mouse (shScr group: n = 5, shSnail group: n = 4). After intraperitoneal injection of luciferin, the mice were imaged with an IVIS Lumina Series III *In Vivo* imaging system (PerkinElmer, Waltham, MA, USA). Live imaging was performed weekly and the bioluminescent images were analysed using Living Image *In Vivo* Imaging Software (PerkinElmer, Waltham, MA, USA) to assess tumour burden at primary and metastatic sites.

### Statistics

Graphical figures and statistical analysis were performed using GraphPad Prism Version 7.0 (GraphPad Software, La Jolla, CA, USA). Detailed information on statistical analysis is described in figure legends.

## Results

### Categorization of HGSOC cell lines by mesenchymal/epithelial status

**Figure 1:**
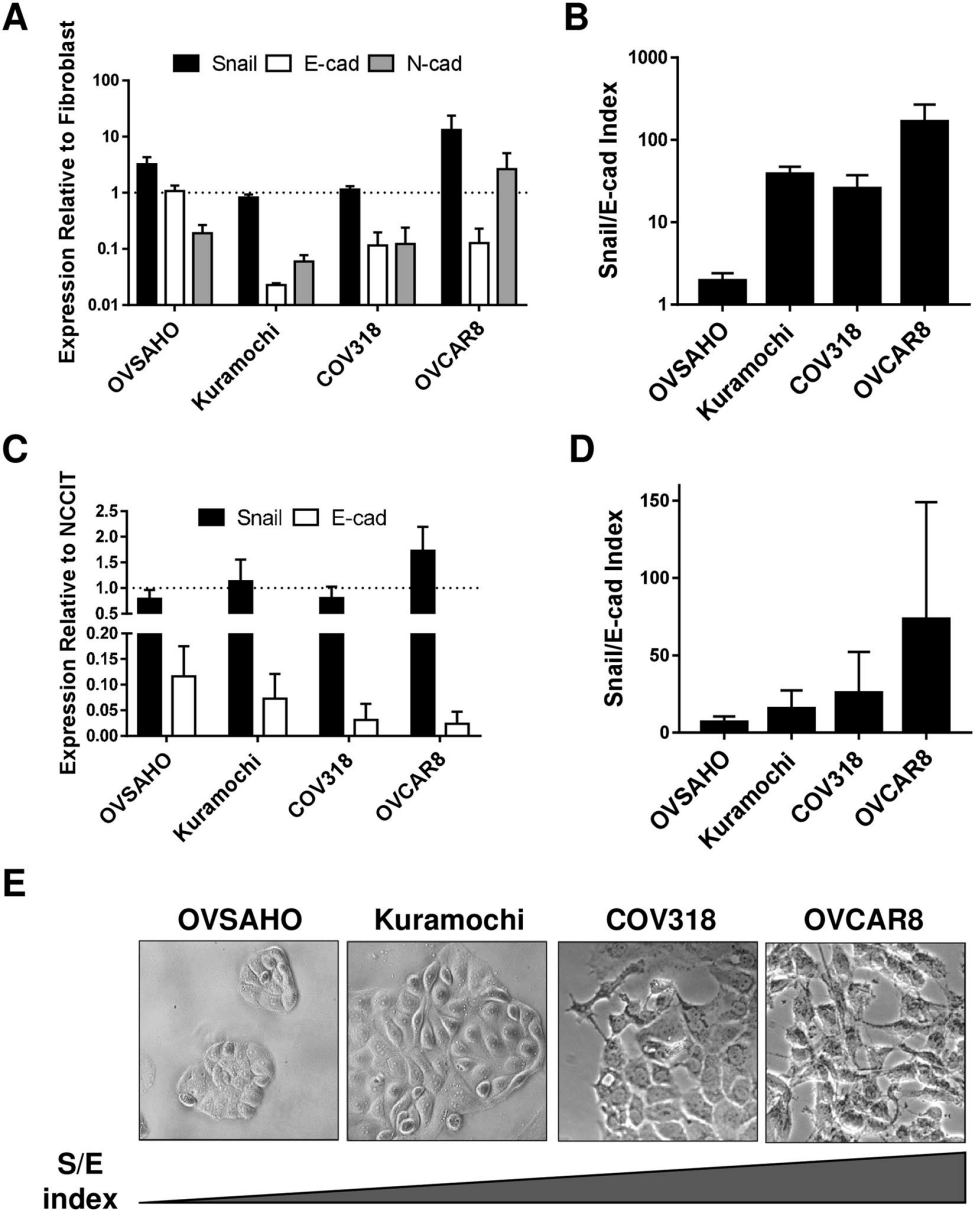
HGSOC cells can be characterized by mesenchymal/epithelial status. (**A**) mRNA expression levels of SNAI1 (Snail; black), CDH1 (E-cad; white), and CDH2 (N-cad; gray bars) in indicated lines relative to fibroblasts by qRT-PCR. β-actin was used for normalization. (**B**) RNA S/E index: qRT-PCR data from A were combined to describe the ratio between SNAI1 and CDH1 mRNA. (**C**) Protein levels of Snail and E-cad from Western blot (WB), normalized to α/β tubulin, relative to NCCIT. For each experiment, all samples were run on a single gel. Gels from independent experiments were analysed in parallel. (**D**) Protein S/E index: WB data from C were combined to describe the ratio between Snail and E-cad. (**A**–**D**) Data represent the mean of at least three independent experiments. Error bars, SEM. (**E**) DIC images of cell lines.

We characterized four of the best HGSOC cell line models, focusing on their epithelial and mesenchymal attributes. RNA and protein levels of Snail, CDH2 and CDH1 varied widely among cell types, and all lines expressed all factors (Fig. 1A,C, Suppl. Figure 1). Because of CDH1 and Snail variability and coexpression, we developed an index that considered relative levels of both factors, reasoning that both should be taken into consideration in determining the functional status of a cell population. On both the RNA and protein level, the Snail/E-cadherin (S/E) index places OVCAR8 at the more mesenchymal end of the spectrum, OVSAHO is most epithelial, and COV318 and Kuramochi are intermediate (Fig. 1B,D). Morphological differences between cell lines corroborated RNA and protein expression; OVCAR8 appeared most mesenchymal, with spindle-shaped cells extending projections, while OVSAHO grew in more epithelial-like tightly-apposed colonies (Fig. 1E).

### Correlation between Snail/E-cadherin index and malignant phenotype

**Figure 2:**
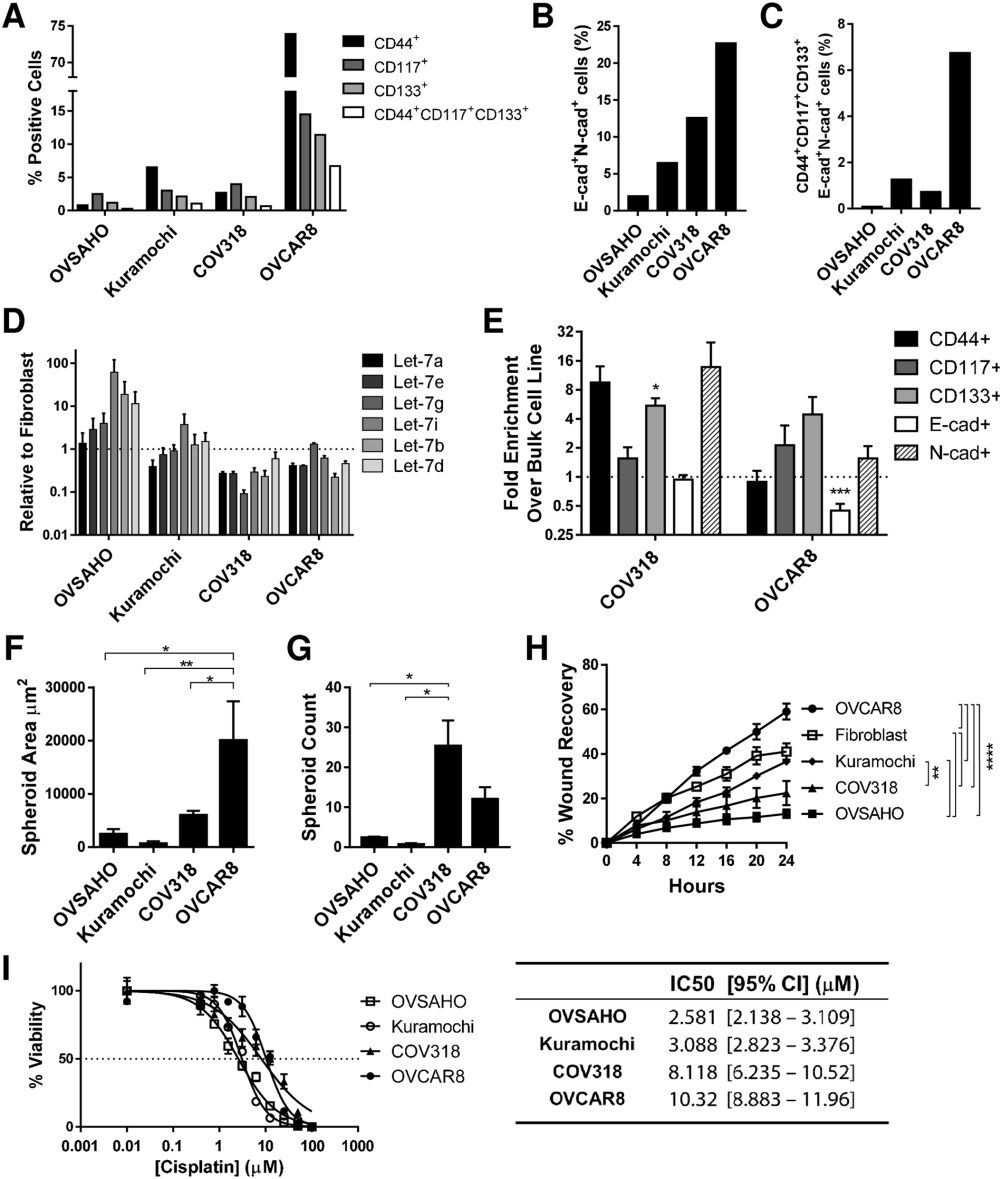
Mesenchymal index in HGSOC cells correlates with malignant phenotype. (**A–C**) Analysis of surface CD44 (black), CD117 (dark grey), CD133 (light grey) and triple positive (white bars) (**A**); E-cad/N-cad double positive cells (**B**); CD44/CD117/CD133/E-cad/N-cad positive cells (**C**) by flow cytometry in indicated cells; shown is frequency in intact cells. (**D**) Levels of let-7 miRNA in indicated lines demonstrated by Taqman qRT-PCR; six let-7 family members are shown. Data are the mean of three independent experiments. Error bars, SEM. (**E**) After culture in spheroid conditions, frequency of cells positive for indicated markers was determined by flow cytometry; fold increase of CD44 (black), CD117 (dark grey), CD133 (light grey), E-cad (white) and N-cad (hatched bars) over bulk cells is shown. Data are the mean of at least three independent experiments. Error bars, SEM; *P < 0.05; ***P < 0.001 by unpaired Student’s t-test. (**F**) Size analysis, and (**G**) numbers of spheroids in indicated cells. Data represent the mean of at least three independent experiments. Error bar, SEM; *P < 0.05; **P < 0.01 by one-way ANOVA with Tukey’s multiple comparison test. (**H**) 24 hour wound healing assays were done on indicated cells; shown are images taken every four hours. Results are the mean ± SEM of three independent experiments. Statistical significance was determined by two-way repeated measures ANOVA with Tukey’s multiple comparison test. **P < 0.0; ****P < 0.0001. (**I**) Chemoresistance assays of cells as indicated. Left panel: x axis, cisplatin concentration; y axis, percent viable cells. Right panel: IC50. Results are the mean ± SEM of triplicate measurement. Curve-fitting and IC50 calculations were carried out with GraphPad Prism software.

We examined the relationship between Snail expression and the malignant phenotype of cell lines. First, the proportion of cancer stem-like cells in each line was evaluated. Using flow cytometry, percentage of cells expressing established ovarian CSC markers CD117, CD133, and CD44^37–39^ was highest in OVCAR8, and lowest in OVSAHO (Fig. 2A, Suppl. Figure 2A). Because hybrid cells have been linked to the stem cell state^23^, we examined the subset of cells positive for both CDH1 and CDH2 (Fig. 2B, Suppl. Figure 2B). These cells comprised between 2% (OVSAHO) and 22% (OVCAR8) of the population; cells positive for these factors and the stem cell markers (CD117, CD133, CD44) were 0.1–6.75% of intact cells (Fig. 2C, Suppl. Figure 3A). Of note, over 95% of the CD117/CD133/CD44 triple positive population in all lines were positive for both CDH1 and CDH2 (OVSAHO 100%, OVCAR8 97.2%), implying that the hybrid phenotype is necessary for the stem cell phenotype (Suppl. Figure 3B). However, between 5% (OVSAHO) and 31.7% (OVCAR8) of the hybrid cells were positive for all three stem cell markers (Suppl. Figure 3B). Thus, the hybrid cells do not all attain the stem cell phenotype.

Because loss of let-7 corresponds with the stem cell state^40^, we determined let-7 levels in the HGSOC cell line panel. Let-7 levels were highest in the more epithelial, and lowest in the more mesenchymal lines (Fig. 2D).

To functionally assess the CSC phenotype, we assessed self-renewal ability of the HGSOC lines via growth in spheroids. Growth in non-adherent spheroid conditions is used as a measure of stemness in both normal^41–43^ and cancer stem cells^38,44–49^. The frequency of cells triple positive for the markers associated with CSC was increased after spheroid culture relative to cells grown in adherent conditions in cell lines assessed (Fig. 2E), confirming that spheroid culture enriches for stem cells. OVCAR8 produced the largest spheroids (Fig. 2F), while COV318 and OVCAR8 produced the greatest number of spheroids (Fig. 2G). Thus, by both let-7 expression and spheroid formation, cells on the mesenchymal end of the S/E ratio spectrum were more stem cell like.

Mesenchymal cells are expected to be more invasive, and indeed a higher proportion of OVCAR8 than OVSAHO cells cross a basement membrane-like barrier^50^. We assessed cell motility of lines by wound healing assay. Rate of wound recovery was greatest in OVCAR8, and least in OVSAHO (Fig. 2H). We examined cisplatin sensitivity by MTT assay. Mesenchymal cells (COV318 and OVCAR8) were more resistant than epithelial cells (OVSAHO and Kuramochi) (Fig. 2I). Taken together, migratory ability and chemoresistance assays revealed that more mesenchymal cells using the S/E index displayed a more malignant phenotype in HGSOC cell lines.

### Knockdown of Snail expression reverses malignant phenotype in HGSOC cells

**Figure 3:**
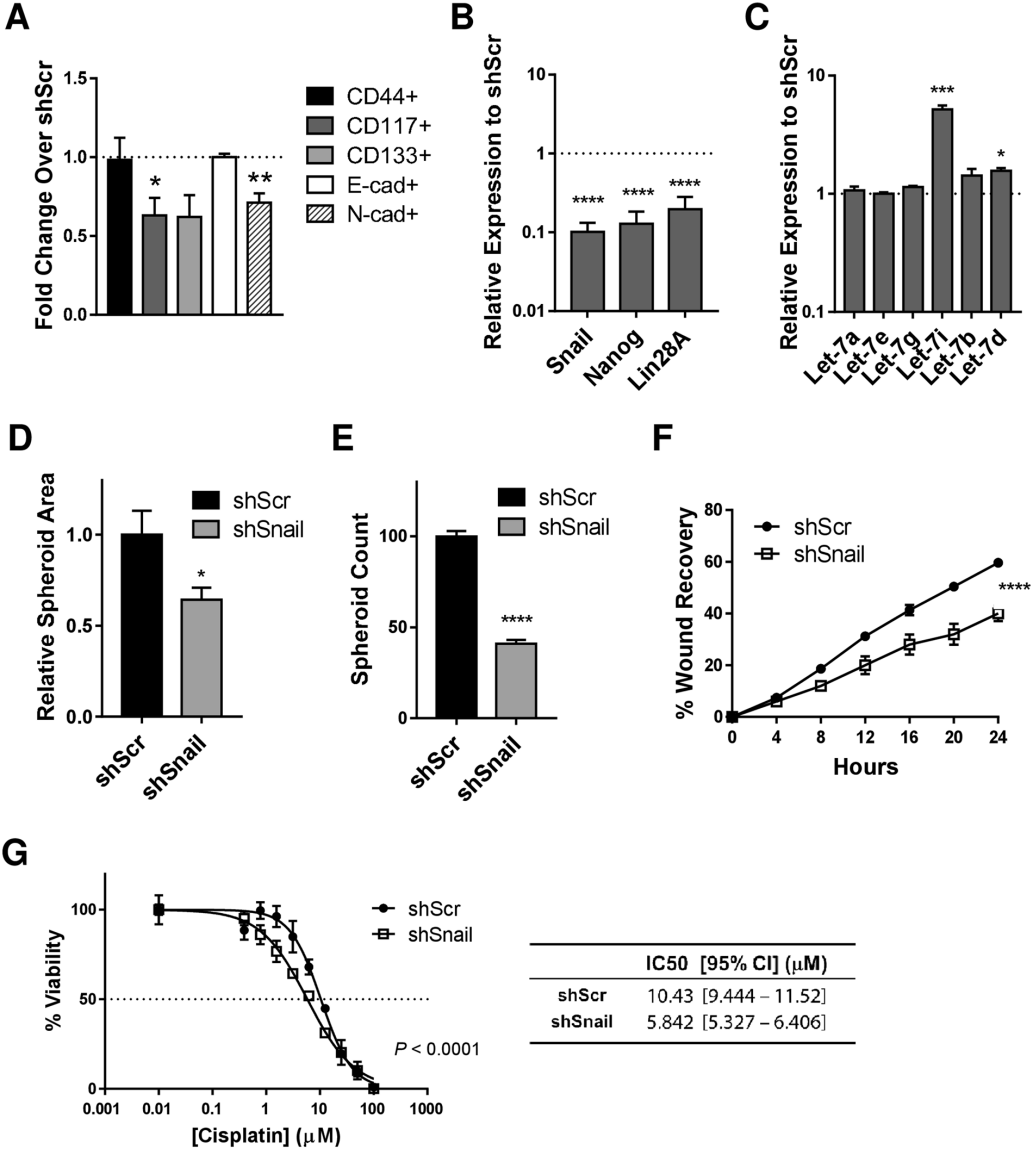
Knockdown of Snail reverses malignant phenotype in HGSOC cells. (**A**) After Snail was knocked down by shRNA, frequency of cells positive for indicated markers was determined by flow cytometry; fold increase of CD44 (black), CD117 (dark gray), CD133 (light gray), E-cad (white) and N-cad (hatched bars) over shScr-transduced control cells is shown. Data represent the mean of three independent experiments. Error bars, SEM; *P < 0.05; **P < 0.01 by unpaired Student’s t-test. (**B**) In Snail knockdown cells, RNA levels of Nanog and Lin28A are reduced, and (**C**) let-7 levels are increased; shown by qRT-PCR. Data represent the mean of at least three independent experiments. Error bars, SEM; ****P < 0.0001 by unpaired Student’s t-test. (**D**) Size analysis, and (**E**) numbers of spheroids in Snail knockdown cells. Results are the mean ± SEM and are representative of at least three independent experiments. Error bars, SEM; *P < 0.05, ***P < 0.0001 by unpaired Student’s t-test. (**F**) Wound healing assay in Snail knockdown (shSnail) and control (shScr) cells. Results are the mean ± SEM of three independent experiments. Statisical significance was determined by two-way repeated measures ANOVA with Sidak’s multiple comparison test. ****P < 0.0001.(**G**) Cisplatin resistance in Snail knockdown (shSnail) and control cells (shScr). Results are the mean ± SEM and are representative of at least three independent experiments. Curve-fitting and IC50 calculations were carried out with GraphPad Prism software.

We next knocked down Snail through the use of virally-delivered shRNA in the most mesenchymal line, OVCAR8 (Suppl. Figures 4,5). We observed that CSC markers CD117 and CD133 both decreased, while CD44 remained unchanged in shSnail relative to shScramble. CDH2 decreased as expected, but CDH1 levels remained unchanged (Fig. 3A). Snail knockdown (KD) resulted in a decrease in expression of Nanog and Lin28 (Fig. 3B), and an increase in let-7 expression (Fig. 3C), both of which are consistent with disruption of the stem cell state. Size and number of spheroids formed with Snail KD was also reduced showing a decrease in self renewal (Fig. 3D,E). Wound healing assays in OVCAR8 cells demonstrated a 30% decrease in migratory ability upon Snail KD (Fig. 3F). Chemoresistance decreased in KD cells (Fig. 3G). These findings demonstrate that inhibiting Snail reverses functional measures of malignant phenotype in HGSOC cells.

### Knockdown of Snail expression decreases tumour burden in an orthotopic xenograft mouse model

**Figure 4:**
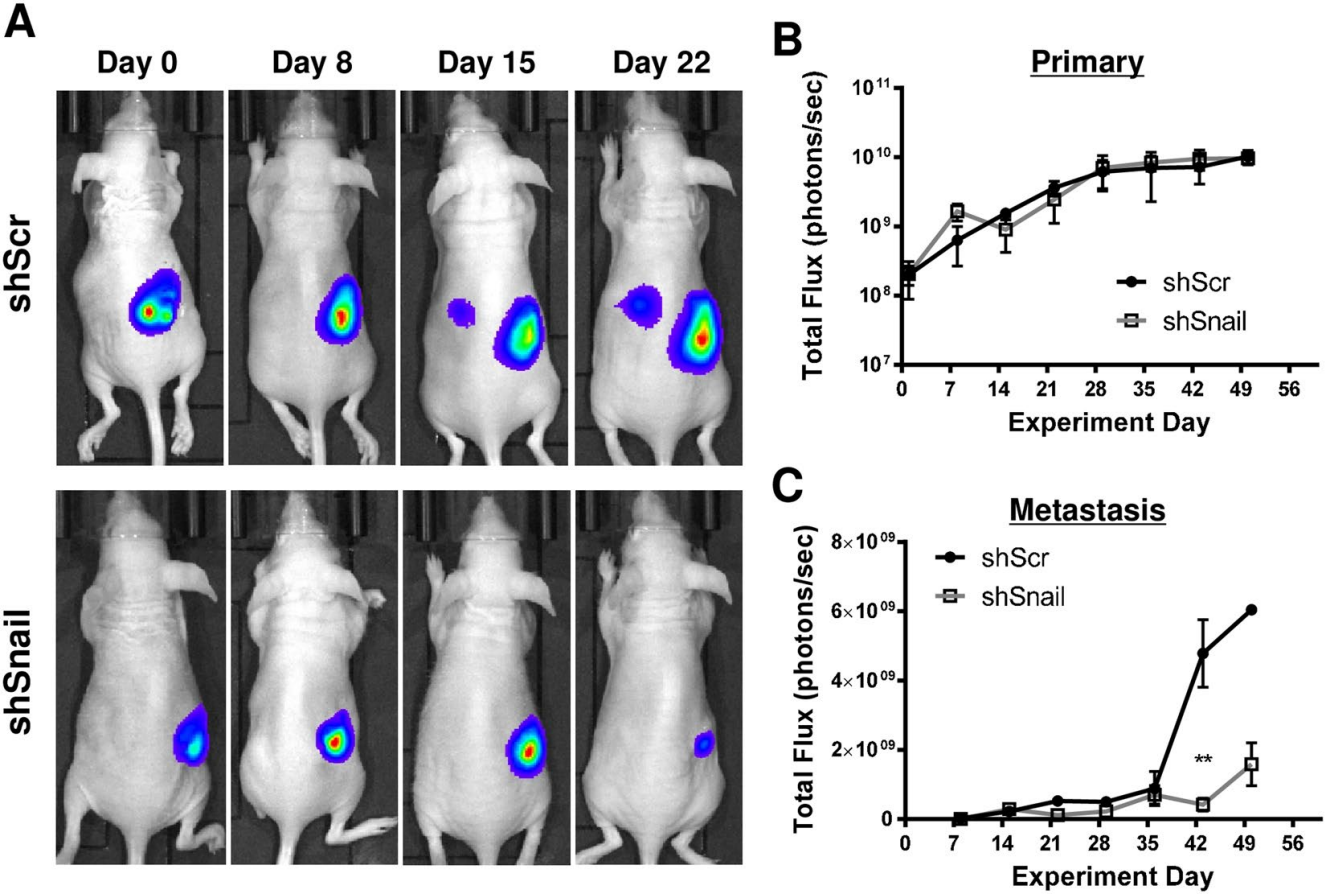
Snail knockdown reduces tumour burden in an orthotopic xenograft mouse model. Luciferized OVCAR8 cells in which Snail (or scrambled control) was knocked down by lentiviral shRNA were injected into the ovarian bursae of Nude mice. Mice were imaged weekly for bioluminescence. (**A**) Representative images of xenograft mice. Control (upper panels) and Snail knockdown (lower panels). (**B**,**C**) Quantitation of bioluminescence at (**B**) primary and (**C**) metastatic sites over seven weeks. X axis, days; y axis, total flux in photons/second; error bars, SEM; **P < 0.01 by two-way repeated measures ANOVA with Sidak’s multiple comparison test.

We chose to develop an orthotopic xenograft model with OVCAR8, known to form clinically relevant tumours in immune compromised mice, while Kuramochi and OVSAHO were shown to be poorly tumorigenic in xenografts^51–53^. Luciferized OVCAR8 cells in which Snail (or scrambled control) was knocked down by lentiviral shRNA were injected into the ovarian bursae of Nude mice in an orthotopic xenograft model. Tumours were observed via bioluminescence imaging weekly for seven weeks, and quantified via total flux. Although primary tumours often appear smaller in shSnail compared to shControl (Fig. 4A), quantitatively they were of similar size (Fig. 4B). This discrepancy may be due to differences in three-dimensional size, or a more compact cellular organization in KD tumours. Metastatic tumours were significantly smaller in mice receiving shSnail cells (Fig. 4C). Upon necropsy, primary and metastatic tumour weights showed a trend toward smaller tumours in shSnail mice (Suppl. Figure 6). Thus, we present evidence that decreasing levels of Snail reduces metastatic tumour burden *in vivo*.

## Discussion

Our studies on acquisition of stemness in reprogramming^25^ led us to hypothesize that factors associated with EMT, specifically Snail, might play pro-stemness roles in HGSOC. We investigated the role of Snail, known to control invasiveness^54^, in HGSOC stemness evolution.

To do these studies, we characterized four of the ovarian cancer lines shown to phenocopy HGSOC samples^29^. One unifying feature of these cells is their lack of a clearly defined epithelial or mesenchymal phenotype. On both RNA and protein levels, all cell populations examined demonstrated the presence of both epithelial and mesenchymal factors (Fig. 1). This may reflect a dysregulation of stereotypical EMT pathways in these cells, or could represent the activity of signals leading to the hybrid state specifically. The hybrid E/M state resulting from partial EMTs has been implicated in both normal development and in cancer^5,21^. There is evidence for linkage between the hybrid state and stemness in both ovarian and breast cancer^21,23^.

Although cells in all of these lines display a hybrid phenotype in that they express both mesenchymal and epithelial markers, comparing levels of Snail and CDH1 allowed us to robustly categorize the lines along the E/M spectrum (Fig. 1). In comparison with more epithelial lines (OVSAHO, Kuramochi) which grow in colonies, the more mesenchymal lines (COV318, OVCAR8) are morphologically more spindle-shaped, and the frequency of cells expressing the established ovarian cancer stem cell surface markers CD133 and CD117 is higher. Culture in spheroid conditions resulted in enrichment of these stem cell markers. Mesenchymal lines formed spheroids more efficiently, further correlating the mesenchymal with the stem cell phenotype. Importantly, cisplatin resistance was higher in the more mesenchymal lines, in agreement with published results^50,53^. Let-7 levels, known to be high in differentiated cells and low in pluripotent stem cells, are lower in the mesenchymal lines COV318 and OVCAR8 (Fig. 2).

Our studies, while not designed to distinguish between stemness markers with regard to prediction of tumourigenesis, do suggest stemness in the CD117/CD133/CD44 triple positive population: it correlated with spheroid-forming ability, and it nearly completely enriched for the E/M hybrid cells (Fig. 2, Suppl. Figure 3)^55^. However, similar to the observations of Strauss *et al*.^21^, the hybrid state is diverse: not all hybrid cells express stem cell markers. Thus, our data are consistent with the hybrid state being necessary, but not sufficient, for the stem cell phenotype. Since the majority of hybrid cells are apparently not stem cells, much remains to be learned about the signalling pathways leading to this state, and cell fate decisions leading to the presence or absence of stemness markers in hybrid cells. Several signalling pathways have been proposed to play roles in partial EMTs, including the balance between Jagged/Notch signalling, regulated by factors such as Numb^56^; and alternative splicing^57,58^.

Inhibiting Snail is associated with an increase in levels of let-7 family members (Fig. 3), critical tumour suppressors with roles in repressing stemness and proliferation. Snail positively regulates Nanog transcription, and associates with Nanog on the protein level leading to direct translational activation of pluripotency genes^8^. The relationships between Let-7, Snail, and pluripotency, and the involvement of Snail in the EMT process, makes Snail an appealing target in cancer research. Our studies suggest a connection between EMT, invasiveness, and traditionally defined stemness markers. These findings serve as *in vitro* basis for a druggable model of tumour progression and chemoresistance in HGSOC.

Expanding our studies to animal models, we demonstrate that Snail inhibition leads to significant reduction of metastatic tumour burden *in vivo* in an orthotopic xenograft model. These data corroborate our cell line findings and provide preclinical evidence for Snail as a feasible target in HGSOC. Further exploration of Snail inhibition in synergy with pharmacologic agents, such as platinum-based chemotherapy and targeted therapies such as PARP inhibitors, are underway.

## Electronic supplementary material

Supplementary Information
