# Supplementary material for: Snail knockdown reverses stemness and inhibits tumour growth in ovarian cancer
Source: Sci Rep. 2018 Jun 7;8:8704. doi: 10.1038/s41598-018-27021-z (PMC5992154; doi:10.1038/s41598-018-27021-z)
Supplement: Supplementary file 1 — Supplementary Information [file 41598_2018_27021_MOESM1_ESM.pdf]

Snail knockdown reverses stemness and inhibits tumour growth in ovarian cancer

Hojo N*[1], Huisken AL*[1], Wang H[1], Chirshev E[1], Kim NS[2], Nguyen SM[3], Campos H[1,4], Glackin CA[5], Ioffe YJ[6], Unternaehrer JJ[1]

1. Division of Biochemistry, Department of Basic Sciences, Loma Linda University, Loma Linda, CA, USA
2. Department of Molecular Biology, Chonbuk National University, Dukjindong 664-14, Jeonju, Jeollabuk-do 561-756, Republic of Korea
3. University of California, Riverside - School of Medicine, Riverside, CA, USA
4. Center for Health Disparities and Molecular Medicine, Loma Linda University, Loma Linda, CA, USA
5. Beckman Research Institute, City of Hope, Duarte, CA, USA
6. Division of Gynecologic Oncology, Department of Obstetrics and Gynecology, Loma Linda University Medical Center, Loma Linda, CA, USA

*Equal contribution

Corresponding author:
Juli Unternaehrer, Ph.D.
Assistant Professor
Department of Basic Sciences
Division of Biochemistry
Loma Linda University School of Medicine
11085 Campus Street
Mortensen Hall 219
Loma Linda, CA 92354
Phone: (909) 558-7691; Fax: (909) 558-4887
junternaehrer@llu.edu

Running title: Snail in ovarian cancer stemness and invasiveness

Keywords: epithelial-mesenchymal transition, stem cells, high grade serous ovarian cancer, metastasis, orthotopic xenograft

Conflict of interest: The authors declare no potential conflicts of interest.

Financial Support: This work was supported by a Grant to Promote Collaboration and Translation from Loma Linda University (LLU) to J.U. and Y.I., and by LLU startup funding. M.G. was supported by a CIRM Bridges grant, H.C. by the Apprenticeship Bridge to College program of the CHDMM

Supplementary Table S1

Primer for	Forward	Reverse
β-Actin (ACTB)	5'-TGAAGTGTGACGTGGACATC-3'	5'-GGAGGAGCAATGATCTTGAT-3'
Snail (SNAI1)	5'-CACTATGCCGCGCTCTTTC-3'	5'-GGTCGTAGGGCTGCTGGAA-3'
E-cadherin (CDH1)	5'-TGCCCAGAAAATGAAAAAGG-3'	5'-GTGTATGTGGCAATGCGTTC-3'
N-cadherin (CDH2)	5'-GAGGAGTCAGTGAAGGAGTCA-3'	5'-GGGAAGTTGATTGGAGGGATG-3'
Nanog	5'-CAAAGGCAAACAACCCACTT-3'	5'-TCTGCTGGAGGCTGAGGTAT-3'
Lin28A	5'-GAGCATGCAGAAGCGCAGATCAAA-3'	5'-TATGGCTGATGCTCTGGCAGAAGT-3'

Supplementary Table 1. Primer sequences used for qRT-PCR.

Supplementary Figure S1

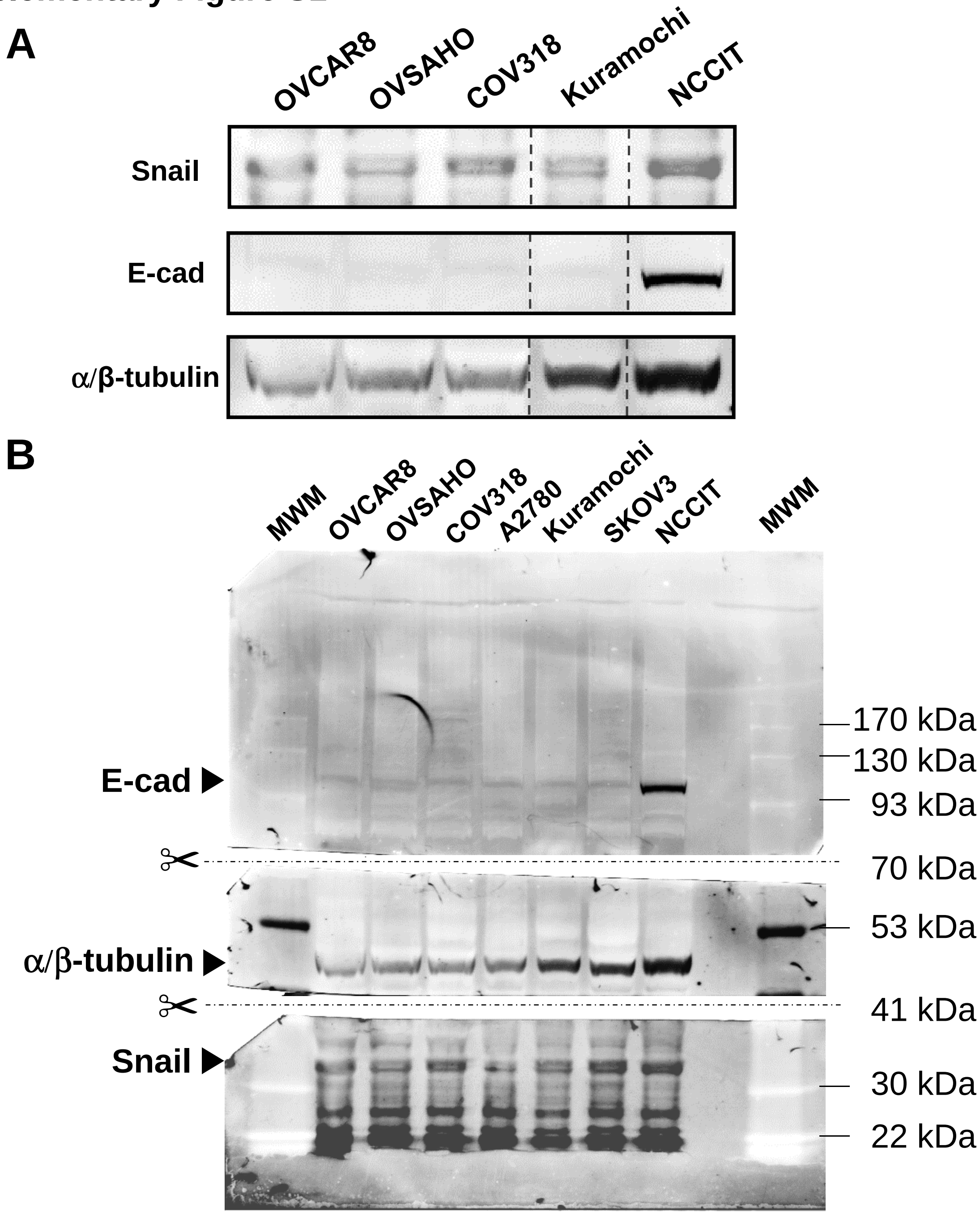

Supplementary Figure S1. Western blot analysis for the quantitation of Snail and E-cadherin protein in HGSOC cell lines. α/β-tubulin was used as housekeeping protein. A. Cropped image. B. Full-length blots of A. After transferring proteins from gel to membrane, the membrane was cut into 3 pieces at the height of 70kDa and 41kDa. Upper, middle, and lower membranes were incubated with anti-E-cad, anti-α/β tubulin and anti-Snail antibodies, respectively.

Supplementary Figure S2

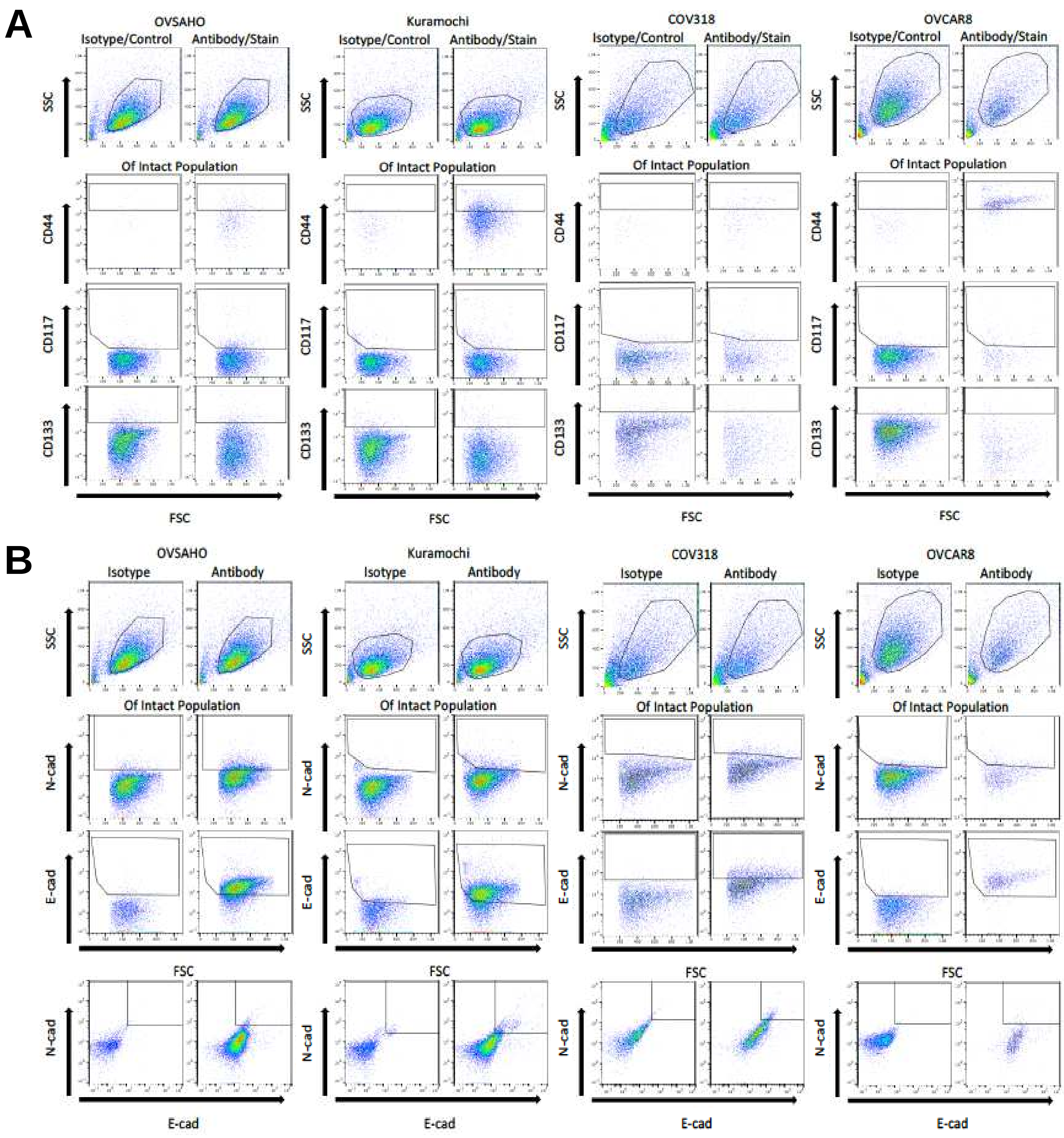

Supplementary Figure S2. Flow cytometry analysis for HGSOC cell lines.
A. Cancer stem cell markers (CD44, CD117, and CD133).
B. Cell surface E-cadherin and N-cadherin. For each antibody, gating was determined based on appropriate isotype-stained controls.

Supplementary Figure S3

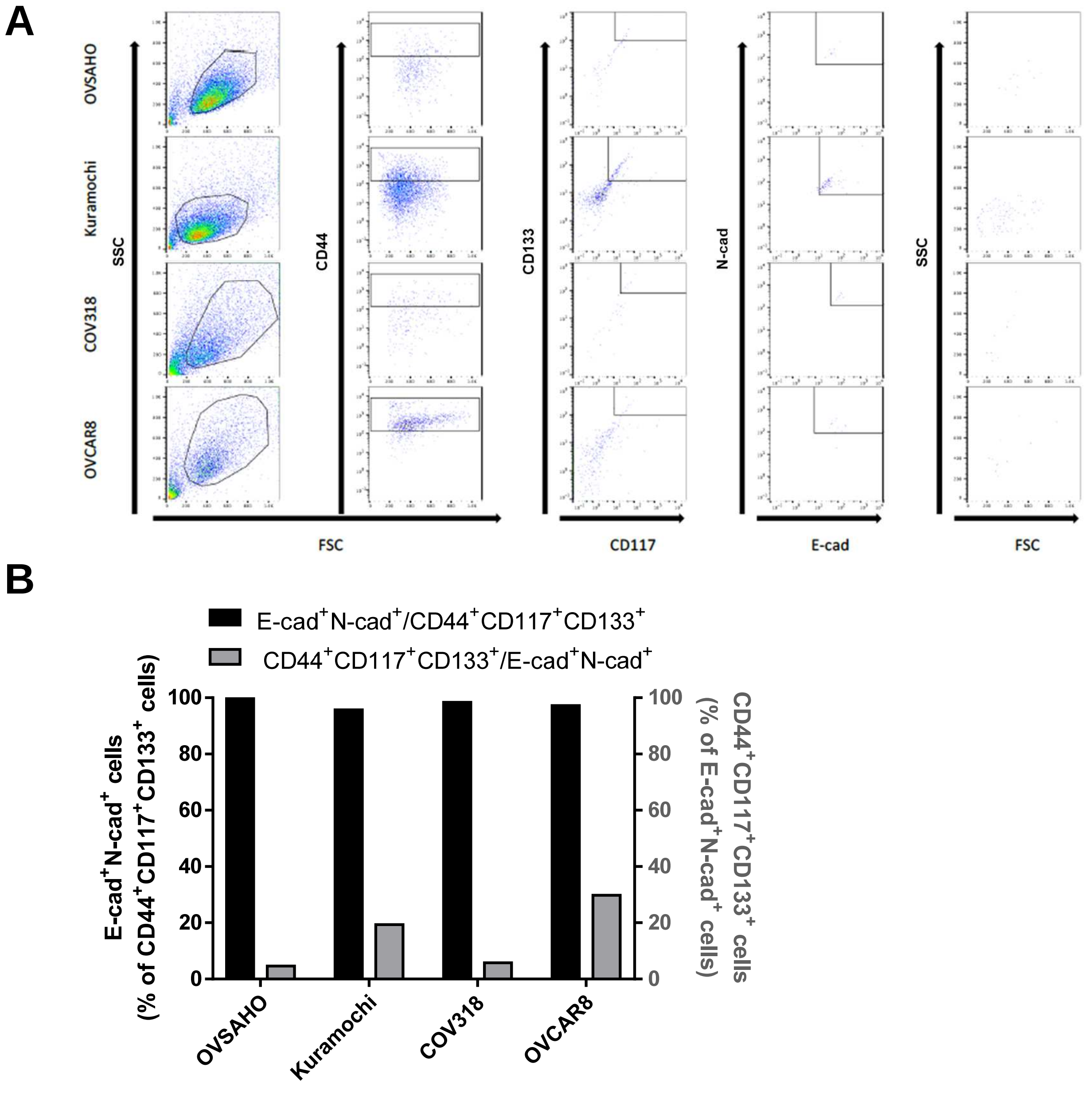

Supplementary Figure S3. Multicolour flow cytometry analysis for HGSOC cell lines.
A. Sequential gating strategy for quantitation of E-cad$^+$N-cad$^+$ cell population in CSC marker positive cell population (CD44$^+$CD117$^+$CD133$^+$).
B. Black bar, percentage of E-cad$^+$N-cad$^+$ cells in CD44$^+$CD117$^+$CD133$^+$ cell population. Grey bar, percentage of CD44$^+$CD117$^+$CD133$^+$ cells in E-cad$^+$N-cad$^+$ cell population.

Supplementary Figure S4

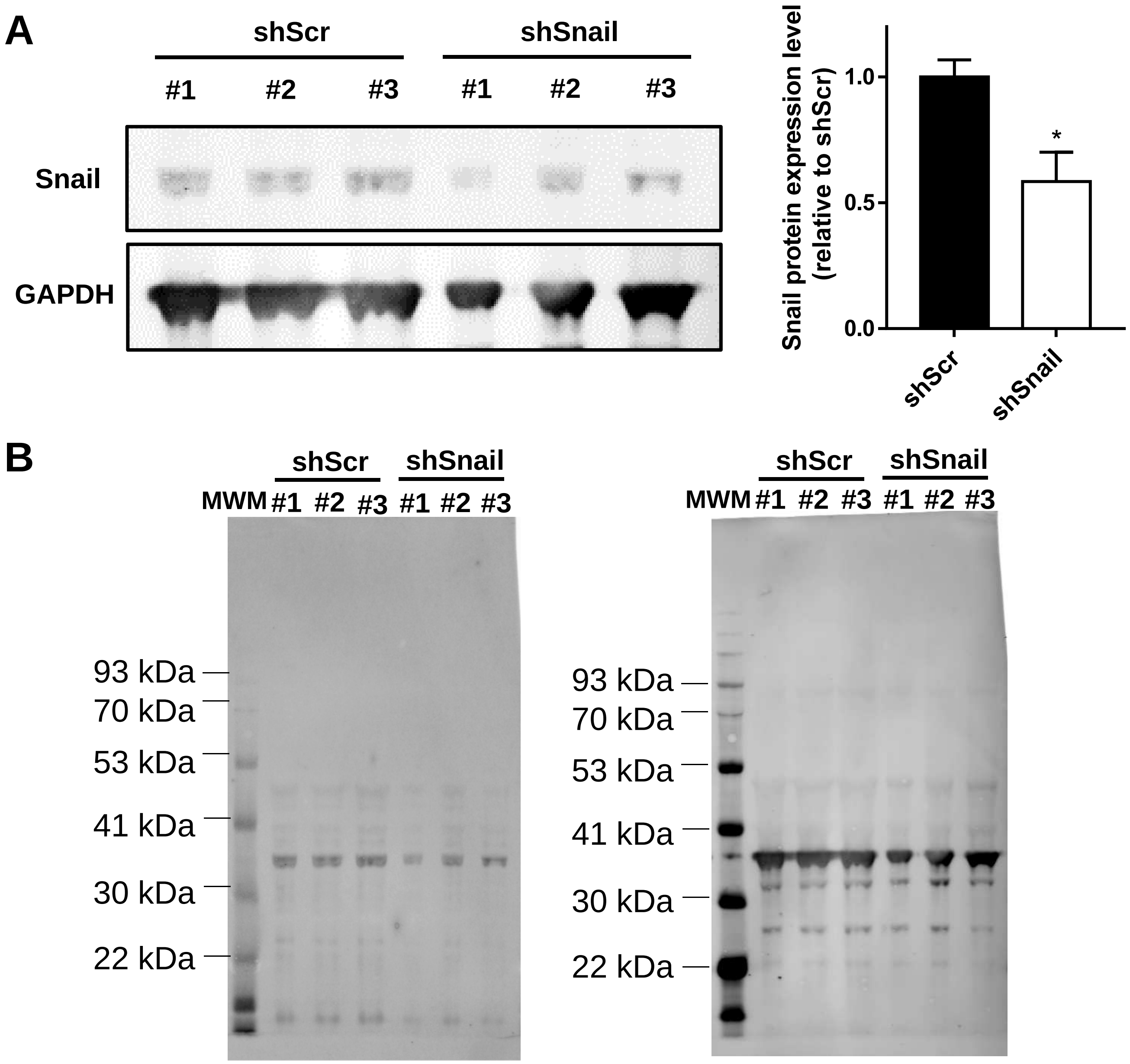

Supplementary Figure S4. Snail knockdown by shRNA in OVCAR8 cells.

A. Left, western blot image for the quantitation of Snail and GAPDH (housekeeping protein). Right, quantification of western blot signals. Data are the mean of biological triplicates. Error bars, SEM; *, $P < 0.05$ by unpaired Student's t-test.

B. Full-length blots of A. The membrane was incubated with anti-Snail mouse antibody and anti-GAPDH rabbit antibody simultaneously. Left, Snail signal was detected by 800nm channel. Right, GAPDH signal was detected by 700nm channel.

Supplementary Figure S5

A

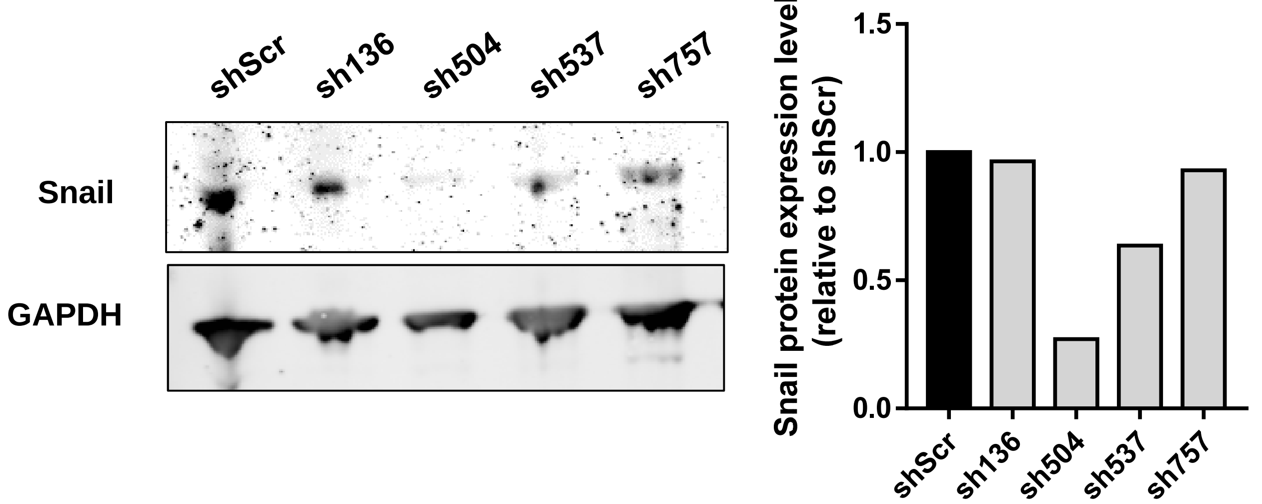

B

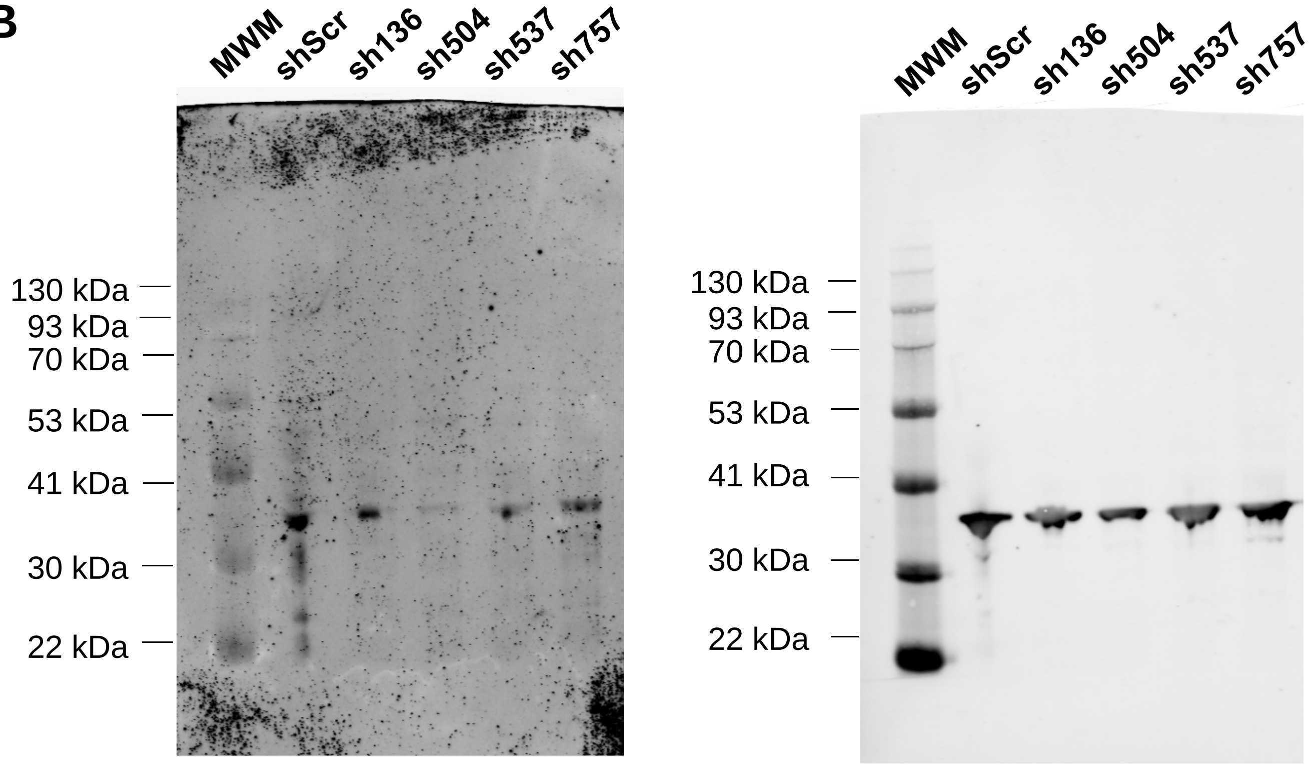

Supplementary Figure S5. Snail knockdown by 4 different shRNAs in OVCAR8 cells.
A. Left, western blot image for the quantitation of Snail and GAPDH (housekeeping protein). Right, quantification of western blot signals.
B. Full-length blots of A. The membrane was incubated with anti-Snail mouse antibody and anti-GAPDH rabbit antibody simultaneously. Left, Snail signal was detected by 800nm channel. Right, GAPDH signal was detected by 700nm channel.

Supplementary Figure S6

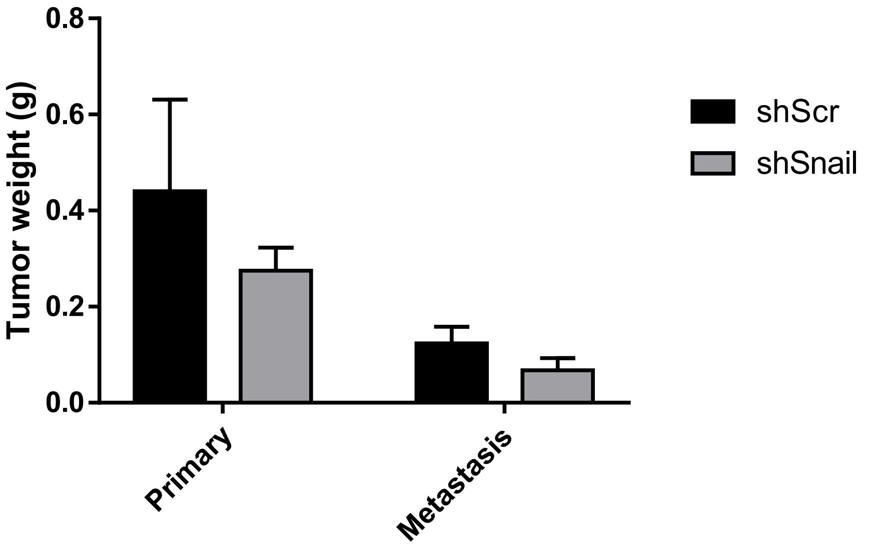

Supplementary Figure S6 Tumour weight in orthotopic xenograft mouse model. Tumour weight in grams for primary and metastatic tumour burden as observed during necropsy. Error bars, SEM.
